# Eye-tracking-aided characterization of saccades and antisaccades in *SYNE1* ataxia patients: a pilot study

**DOI:** 10.1186/s12868-021-00612-9

**Published:** 2021-02-01

**Authors:** Laszlo Szpisjak, Gabor Szaraz, Andras Salamon, Viola L. Nemeth, Noemi Szepfalusi, Gabor Veres, Balint Kincses, Zoltan Maroti, Tibor Kalmar, Malgorzata Rydzanicz, Rafal Ploski, Peter Klivenyi, Denes Zadori

**Affiliations:** 1 Department of Neurology, University of Szeged, Semmelweis u. 6, 6725 Szeged, Hungary; 2 Department of Psychiatry, University of Szeged, Szeged, Hungary; 3 Genetic Diagnostic Laboratory, Department of Pediatrics and Pediatric Health Center, University of Szeged, Szeged, Hungary; 4 Department of Medical Genetics, Medical University of Warsaw, Warsaw, Poland; 5 MTA-SZTE Neuroscience Research Group, Szeged, Hungary

**Keywords:** *SYNE1*, Ataxia, Genetics, Eye movement, Eye tracking, Saccade

## Abstract

**Background:**

*SYNE1* ataxia is an autosomal recessive hereditary condition, the main characteristic features of which are gait and limb ataxia and cerebellar dysarthria. Reports have revealed that the clinical phenotype of *SYNE1* ataxia is more complex than the first published cases with pure cerebellar signs indicated. The aim of this study was to characterize eye movement alterations in the first diagnosed Hungarian *SYNE1* ataxia patients.

**Results:**

Saccades and antisaccades were examined with an eye tracker device in 3 *SYNE1* (one patient has two frameshift mutations [c.8515_8516insA, p.Met2839Asnfs*53 and c.11594_11595insG, p.Glu3866*] in a compound heterozygous state, whereas two subjects have a splicing variant [c.23146-2A > G] in a homozygous state), 6 Friedreich ataxia (FA) patients and 12 healthy controls. Besides that, detailed clinical phenotyping and comprehensive neuropsychological assessment were carried out in all patients with ataxia.

In addition to the characteristic cerebellar alterations, pyramidal signs and polyneuropathy were observed at least in 2 *SYNE1* ataxia patients, for which no other underlying reason was found. The eye tracking assessment revealed hypometric saccades in the longer amplitude (18.4°) saccadic paradigm in all *SYNE1* patients, whereas 2 out of 3 *SYNE1* subjects performed slow saccades as well. In the antisaccade task, higher incorrect ratios of antisaccades were demonstrated in *SYNE1* patients compared to healthy controls, showing inverse correlation with working memory test results. The corresponding data of FA patients was dispersed over a wide range, partially overlapping with control data.

**Conclusions:**

The current study draws attention to the presence of eye movement disorders in patients with *SYNE1* ataxia and demonstrates that alterations in the antisaccade paradigm may be related to working memory deficits.

## Background

Autosomal recessive cerebellar ataxias (ARCA) belong to a continuously expanding group of hereditary neurodegenerative disorders. Recently, more than 100 genes have been identified which can cause ARCA, including the *SYNE1* gene (OMIM 608,441). *SYNE1* is one of the largest genes in the human genome, located in 6p25 chromosome and containing 146 exons [1]. This huge gene encodes a peptide of about 8797 amino acids, known as Nesprin 1 (Nuclear envelope spectrin 1) [1]. It is a member of the spectrin family of proteins and its major function is to link the plasma membrane to the actin cytoskeleton [2]. Nesprin 1 has three domains, including the N-terminal actin binding domain (also called calponin homology domain), multiple spectrin repeats and the C-terminal KASH domain (also knowns as Klarsicht domain) [1]. In 2007, Gros-Louis et al*.* reported 26 French-Canadian families from Quebec, Canada with slowly progressive pure cerebellar hereditary ataxia caused by truncating mutations of the *SYNE1* gene. The name of this disease was autosomal recessive cerebellar ataxia type 1 (ARCA1), also known as spinocerebellar ataxia, autosomal recessive 8 (SCAR8), or recessive ataxia of Beauce [1]. In the following years, *SYNE1* ataxia was observed almost exclusively in Quebec, Canada [1, 3]. From 2013, some sporadic cases were reported outside the French-Canadian population as well [4–7]. In 2016, Synofzik and Mademan et al*.* described 33 non-Canadian patients with *SYNE1* ataxia from a large multi-center study, which indicated that mutations of *SYNE1* gene are much more common causes of ARCA than previously thought [2, 8]. Besides its frequency, the clinical phenotype was also more complex than the first described, purely cerebellar disease. Most of the newly identified patients had extracerebellar neurological signs, including upper and lower motoneuron symptoms, and non-neurological abnormalities, including scoliosis, pes cavus or respiratory dysfunction with severe manifestation. Only a small portion of these subjects showed the classical pure cerebellar phenotype [2, 8].

Moreover, mutations of *SYNE1* gene have been associated with arthrogryposis multiplex congenita, Emery-Dreifuss muscular dystrophy 4, dilatative and hypertrophic cardiomyopathy, intellectual disability, blepharospasm, autism spectrum disorder and schizophrenia [9–16].

After reviewing the clinical phenotype of the previously published 168 *SYNE1* ataxia patients, it was noted that detailed characterization of eye movements had not yet been performed, only the occurrence of gaze-evoked nystagmus, slowing of saccades, broken up smooth pursuits, strabismus and square-wave jerks were reported [1–4, 17–19].

In this paper we aimed to characterize the saccadic and antisaccadic eye movements of 3 Hungarian *SYNE1* ataxia patients and compare them to the same parameters of Friedreich ataxia (FA) patients and healthy subjects in addition to detailed clinical phenotyping and comprehensive neuropsychological assessment.

## Patients and methods

### Participants

9 patients with unknown cerebellar ataxia and 12 healthy controls (HC) were enrolled in the study. The patients underwent a detailed diagnostic approach including neurological examination, laboratory and radiological investigations to exclude acquired causes of ataxia. Scale for the Assessment and Rating of Ataxia (SARA) scores were recorded in all cases. After obtaining written, informed consent, genomic DNA was extracted from peripheral blood leukocytes by standard protocol. First, according to recent guidelines on the management of sporadic ataxias without known secondary etiology [20], the most common repeat expansion hereditary ataxias (spinocerebellar ataxia (SCA) 1, 2, 3, 6, 7 and FA) were tested. If these genetic tests did not confirm the diagnosis, new generation sequencing (NGS) was performed.

For proband AT-04, whole exome sequencing (WES) was performed with SureSelectXT Human kit All Exon v7 (Agilent, Agilent Technologies, Santa Clara, CA) according to the manufacturer’s instructions and paired-end sequenced (2 × 100 bp) on HiSeq 1500 (Illumina, San Diego, CA, USA). Prioritized variants were validated in the proband, in the parents of the proband and in his brother by amplicon deep sequencing performed using Nextera XT Kit (Illumina) and sequenced on HiSeq 1500 (Illumina).

For subjects AT-05 and AT-06, a total of 60 ng of genomic DNA was used for library preparation and sequenced with Trusight One clinical exome kit (Illumina) on Illumina MiSeq platform. The clinical exome kit covers the coding region of 4813 clinically relevant, disease-associated genes. The 150 bp paired reads were aligned to the GRCh37.75 human reference genome by Burrows Wheel Aligner (BWA v0.7.9a) software. The variants were called by Genome Analysis Toolkit HaplotypeCaller (GATK v3.5) best practice; annotated by SnpEff and VariantStudio softwares. Variants were filtered based on severity and frequency against public variant databases, including dbSNP, ClinVar, ExAC, EVS and an in-house clinical exome database of 140 unrelated Hungarian patients.

### Eye tracking

#### Recording system

The system and paradigm that were used are described in a previous study [21]. The assessment was performed in a well-lit room. Subjects sat in front of the monitor and their heads were fixed at a distance 60 cm from the screen. We used a Tobii TX300 eye tracker and tasks were programmed in Psychophysics Toolbox V 3.0.12, under MatLab. Before every paradigm, a five-points calibration was performed.

#### Saccade task

Subjects accomplished the following visually guided saccade task: a black cross appeared at the center of the screen and 1.2–2 s later it jumped to the right or left side of the screen. The background was grey and the distances of displacement of the cross were 9.2*°* or 18.4*°* horizontally. All measurements were repeated 20 times in a pseudorandom order, this means 80 measurements per subject. The participants had to shift their gaze to the new position of the target as fast and accurately as they could. There was a break half-way through the task to prevent subjects tearing and/or tiring.

#### Antisaccade task

In the antisaccade task, the simple antisaccade paradigm was used [22]. The composition was similar to the visually guided saccade paradigm, however, the participants had to direct their gaze in the opposite direction (e.g. if the target appeared on the left side, they had to look to the right side). We explained explicitly the antisaccade paradigm to the patients before the task and answered their questions. We particularly highlighted for the participants that the antisaccade task needs more attention. Before the trial, all patients confirmed that they understood the task instructions. Only horizontal movements were recorded, as in the saccade task. There was also a break after the first half of the trial.

#### Data acquisition and processing

Data recording began when the target jumped to the periphery and stayed there for one second. The recording frequency was 300 Hz and both eyes were registered separately. We used a semi-automatic, in-house script to define parameters of saccades, as described in a previous study [21]. The following parameters were measured: peak velocity, latency, amplitude, gain and duration. In the saccade task, we assessed the main sequence relationships of duration versus amplitude and peak velocity versus amplitude using the linear model [23]. Additionally, in the antisaccade paradigm the incorrect ratio of antisaccades was also examined. It is a quotient showing the incorrectly executed antisaccades, calculated as incorrect/(incorrect + correct) antisaccades.

### Neuropsychological assessment

The enrolled ataxia patients were assessed via cognitive examination performed by trained neuropsychologists. The global cognitive performance was measured by Addenbrooke’s Cognitive Examination (ACE) including the Mini-Mental State Examination (MMSE). Executive function was evaluated by verbal and semantic fluency tests. In addition, working memory and the ability to maintain and manipulate information were estimated by the Backward Digit Span Task (BDST) and the Listening Span Task (LST). The quality of information planning and visuo-constructional and visual organizational abilities were assessed by the Rey Complex Figure Test (RCFT).

## Results

### Patients

**Fig. 1:**
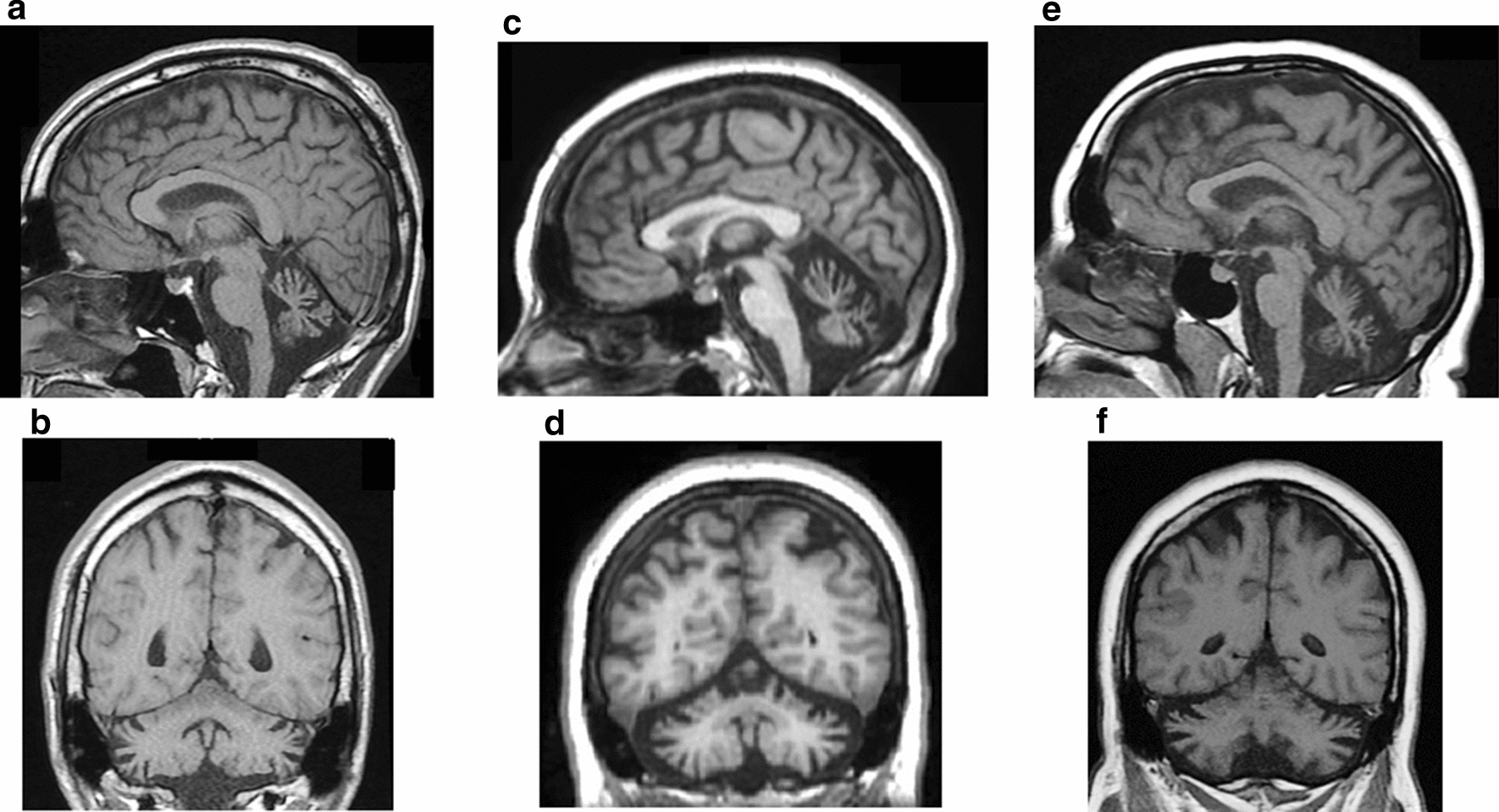
Brain MRI scans of *SYNE1* ataxia patients demonstrated moderate cerebellar atrophy in all subjects and mild cortical atrophy in AT-05 and AT-06 patients. **a**, **b**: AT-04 patient; **c**, **d**: AT-05 patient; **e**, **f**: AT-06 patient; **a**, **c**, **e**: sagittal T1 weighted scans; **b**, **d**, **f**: coronal T1-weighted scans

The repeat expansion examinations verified the FA diagnosis of 6 patients. All of them had homozygous GAA repeat expansions in the first intron of the *FXN* gene. The remaining three patients had negative repeat expansion tests, therefore NGS was performed and it confirmed *SYNE1* gene abnormalities. The mean age of FA patients and HC group participants was the same, and the three *SYNE1* patients were in a similar age range. The demographic and clinical data of FA and *SYNE1* patients and healthy subjects are summarized in Table 1, while the thorough clinical and genetic characteristics of *SYNE1* patients are detailed here. AT-04 subject was the second child of Hungarian, non-consanguineous parents. There was no neurological disease in his family. His first symptom was gait ataxia at the age of 15 years. He also had delayed puberty in this period. Later, slurred speech also appeared and his gait imbalance progressed. The neurological examination revealed gaze-evoked horizontal nystagmus, cerebellar dysarthria, bilateral Babinski sign, gait ataxia and severe lower limb ataxia and mild numbness in the upper extremities. Sometimes stimulus sensitive myoclonic jerks could also be observed. He had strabismus and myopia with negative fundoscopy. Electroneurography showed mild axonal sensory polyneuropathy. Currently, the patient requires walking sticks because of the progression of his symptoms. Laboratory examination did not find pathological abnormalities. Brain MRI was performed after sixteen years of disease course and displayed moderate cerebellar atrophy with preserved brainstem and supratentorial structures (Fig. 1a, b).

**Table 1:** Demographic, clinical and genetic data of *SYNE1* and FA ataxia patients and healthy controls

Patient code/Group name	Age (years)	Sex	Mutation (cDNA)	Protein change or variant type	Age at onset (years)	Gait ataxia	Upper limb ataxia	Lower limb ataxia	Dys-arthria	GEN	UMN	LMN	PNP	SARA	Brain MRI	Other sign/ disease
AT-04	Mean: 38.3 ± 3.40 (35–43)	M	c.8515_8516insA	p.Met2839Asnfs*53	15	+ + +	+ +	+ + +	+ +	Y	Y	N	Mild ASN	23.5	Cerebellar atrophy	Delayed puberty, myoclonic jerks, myopia, strabism
			c.11594_11595_insG	p.Glu3866*												
AT-05		F	c.23146-2A > G	Splicing	30	+ + +	+ +	+ + +	+ +	N	Y	N	N	25	Cerebellar and cerebral cortical atrophy	DM, HC, HT, obesity
AT-06		F	c.23146-2A > G	Splicing	14	+ +	+	+	+	N	Y	N	MSMN	12	Cerebellar and cerebral cortical atrophy	DM, HC, HT, obesity, pes cavus
FA group	Mean: 41.5 ± 17.97 (16–60)	3 M, 3F	Homozygous intronic GAA repeat repeat expansion	Decreased frataxin expression	Mean: 25.83 ± 16.64 (7–49)	+ + 5/6 + + + 1/6	+ 4/6 + + 2/6	+ 1/6 + + 4/6 + + + 1/6	+ 1/6 + + 4/6 + + + 1/6	N 6/6	Y 5/6 N 1/6	Y 1/6 N 5/6	ASN 2/6 ASMN 1/6 NSP 2/6 N 1/6	Mean: 16 ± 6.5 (13–30.5)	–	–
HC group (ST)	Mean: 40.0 ± 10.58 (28–59)	4 M, 8F	–	–												
HC group (AST)	Mean: 40.25 ± 10.39 (28–59)	4 M, 8F	–	–												

+ : mild, +  + : moderate: +  +  + : severe

*ASMN *axonal sensorimotor polyneuropathy, *ASN *axonal sensory polyneuropathy, *AST* antisaccade task, *DM* diabetes mellitus, *F* female, *GEN* gaze-evoked nystagmus, *HC* hypercholesterolemia, *HT* hypertension, *M* male, *MSMN* mixed sensorimotor polyneuropathy, *N* not present, *NSP* not specified polyneuropathy, *PNP* polyneuropathy, *SARA* Scale for the Assessment and Rating of Ataxia, *ST* saccades task, *UMN* upper motor neuron involvement, *Y* present

**Fig. 2:**
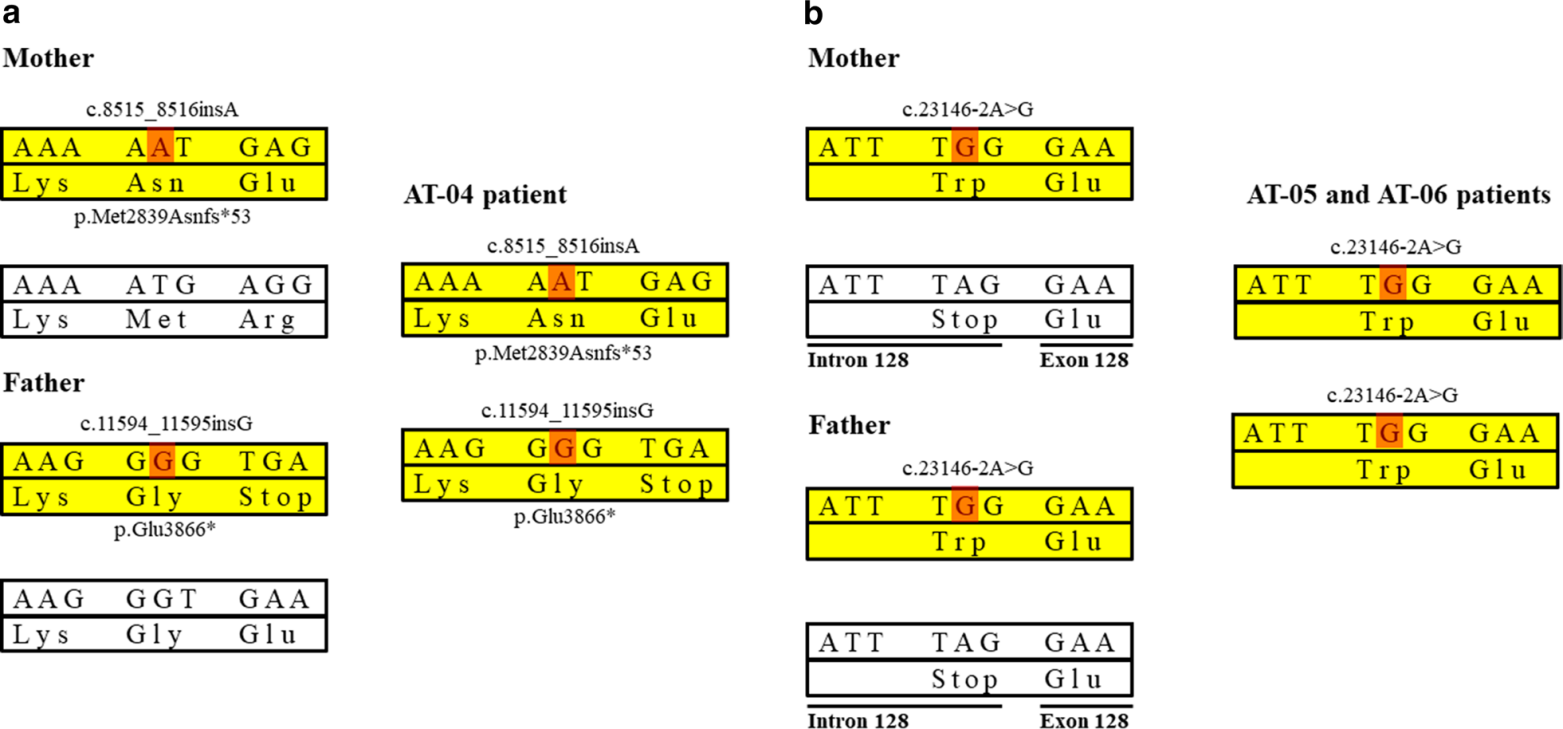
Genetic abnormalities and consequent alterations of protein of *SYNE1* ataxia patients and their parents. **a**: *SYNE1* gene mutations in AT-04 patient and the parental origin of these variations. **b**: *SYNE1* gene abnormalities in AT-05 and AT-06 subjects and the parental segregation of these mutations. The upper parts of the bars denote the DNA sequence, while the lower parts show the encoded amino acids of the protein. Yellow bars indicate the pathogenic alleles, white bands mark the normal alleles. Red highlights the nucleotide change of the *SYNE1* gene. In part (**b**), the c.23146-2A > G mutation is located in the intron–exon boundary resulting in an abnormal splicing variant

WES of AT-04 patient revealed a compound heterozygote state in *SYNE1* gene NM_033071.3:c.8515_8516insA, p.Met2839Asnfs*53 and NM_033071.3:c.11594_11595insG, p.Glu3866* (Fig. 2a). The c.8515_8516insA variant located in exon 55 out of 146 was inherited from the mother of the proband, while c.11594_11595insG located in exon 71 was inherited from the father, and both variants were absent in the healthy brother of the proband. None of the frameshift variants were found in the gnomAD database (www.gnomad.broadinstitute.org) and they are predicted to cause the loss of the full-length SYNE1 protein (8750 amino acids).

The age at onset of AT-05 patient was 30 years and her first complaint was gait ataxia, whereas the first symptom of her sister (patient AT-06) appeared at 14 years of age and was gait abnormality as well. The neurological examination of both patients revealed cerebellar dysarthria and brisk tendon reflexes with bilateral Babinski signs. Truncal ataxia was moderate in the younger patient (AT-06) and was severe in the elder subject (AT-05). After eleven years of disease course patient AT-05 could only walk with aids. Mild upper limb and moderate lower extremity incoordination developed in the younger sister, whereas her sibling had moderate superior and severe inferior limb ataxia. AT-05 patient has obesity, diabetes mellitus, hypertension and hypercholesterolemia, but ophthalmological and cardiological investigations were normal. AT-06 patient also has the same metabolic disorders, moreover, she has an excavated foot and electroneurography delineated multifocal sensorimotor mixed type polyneuropathy. The brain MRI showed moderate cerebellar and very mild cerebral cortical atrophy in both patients (Fig. 1c–f). Their non-consanguineous parents did not suffer from ataxia and the younger patient has two healthy children.

In AT-05 and AT-06 patients the same homozygous NM_182961.3:c.23146-2A > G alteration of the *SYNE1* gene was detected. This intronic variant was not found in gnomAD. It causes a TAG–TGG codon change at the Intron 128 – Exon 128 boundary resulting in an abnormal splicing variant (Fig. 2b). The presence of these mutations was confirmed by targeted Sanger sequencing. Segregation analysis identified this variant in the heterozygous state in both parents of the patients.

### Eye tracking

#### Saccades

**Fig. 3:**
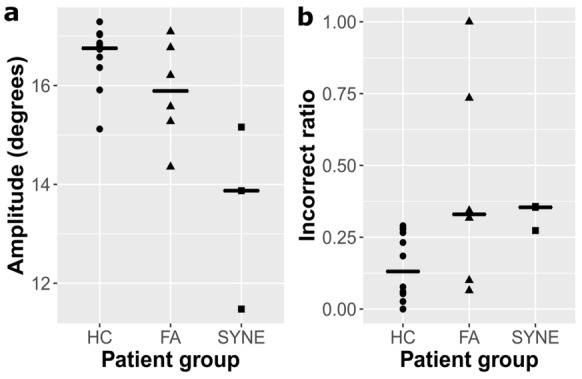
The most characteristic alterations in saccadic and non-saccadic paradigms in *SYNE1* ataxia patients. **a**: saccadic amplitude of the 18.4 saccade paradigm in the different groups; **b**: incorrect ratios of the 18.4 antisaccade task in the investigated subjects; the circles, triangles and squares denote the parameters of healthy controls (HC), Friedreich ataxia patients (FA) and *SYNE1* patients, respectively, and the median values are demonstrated as well

**Fig. 4:**
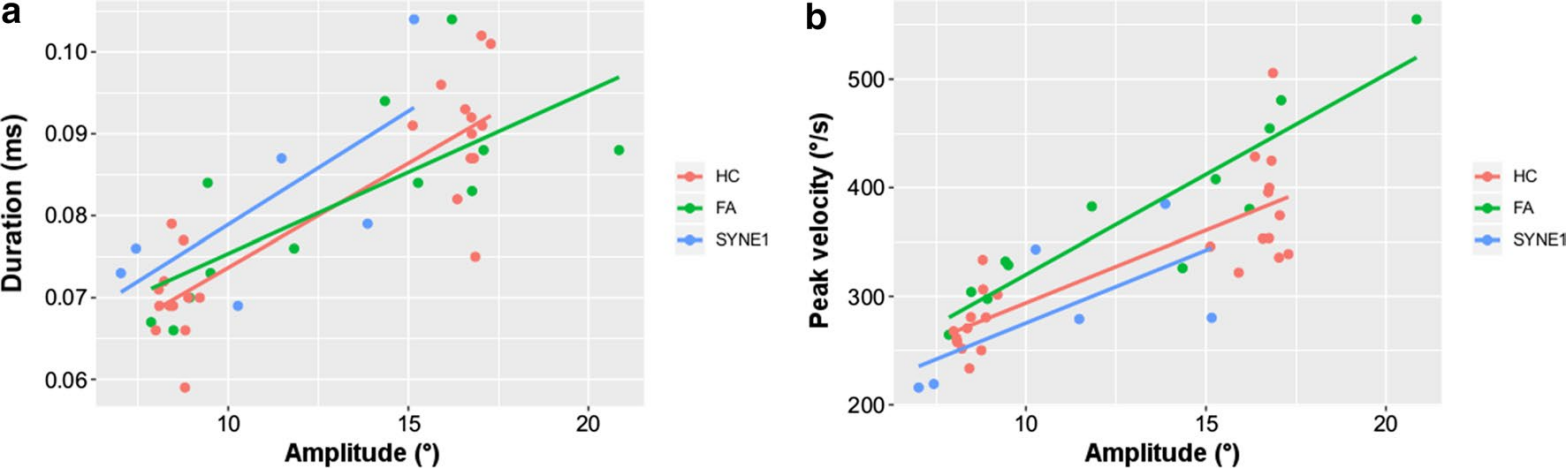
The main sequence relationships of saccades using the linear model. **a**: saccadic duration versus amplitude; **b** saccadic peak velocity versus amplitude; the red, green and blue dots denote the parameters of healthy controls (HC), Friedreich ataxia patients (FA) and *SYNE1* subjects, respectively

The pooled data of leftward and rightward saccades were analyzed (Table 2). There was not any relevant difference between the three groups of examined subjects in saccadic latencies and durations for either the shorter (9.2°) or the longer (18.4°) saccade paradigms. The peak velocities of saccades of AT-05 and AT-06 patients were smaller than the HC subjects and FA patients. However, the peak velocities of the saccades of AT-04 patient were similar to the subjects of HC and FA groups. In the 9.2° saccade task, AT-04 patient demonstrated hypermetric saccadic eye movements, whereas the other two *SYNE1* ataxia patients showed hypometric saccades. Nevertheless, in the 18.4° saccade task *SYNE1* ataxia subjects performed smaller saccadic amplitudes and gain than the healthy controls with minimal overlap (Fig. 3a). The amplitudes and gain of saccades of FA patients were in a similar range to that of the HC group. Figure 4 displays the main sequence relationships using the linear model. The duration vs. amplitude diagram (Fig. 4a) shows that saccades of *SYNE1* ataxia patients are hypometric and their duration is longer than in FA or HC groups. The peak velocity vs. amplitude graph (Fig. 4b) reinforces that the saccades of *SYNE1* patients are hypometric and their peak velocity is smaller than in HC or FA groups.

**Table 2:** Saccade examination in *SYNE1* (AT-04–06) and Friedreich ataxia patients and in healthy controls

Subjects	9.2° saccades					18.4° saccades				
	Peak velocity (°/s)	Latency (s)	Amplitude (°)	Duration (s)	Gain	Peak velocity (°/s)	Latency (s)	Amplitude (°)	Duration (s)	Gain
AT-04	343.18	0.16	10.27	0.069	1.117	384.95	0.19	13.87	0.079	0.754
AT-05	219.14	0.27	7.434	0.076	0.808	279.08	0.27	11.48	0.087	0.624
AT-06	215.87	0.18	7.02	0.073	0.763	280.26	0.22	15.16	0.104	0.824
Median FA (range)	316.45 (264.62–382.63)	0.20 (0.18–0.31)	9.18 (7.86–11.83)	0.071 (0.066–0.084)	0.998 (0.855–1.285)	431.13 (326.02–555.22)	0.23 (0.21–0.34)	16.49 (14.35–20.85)	0.088 (0.083–0.104)	0.896 (0.780–1.133)
Median HC (range)	269.20 (233.54–333.55)	0.18 (0.17–0.21)	8.45 (7.99–9.21)	0.069 (0.059–0.079)	0.919 (0.869–1.001)	363.93 (321.89–505.69)	0.20 (0.17–0.26)	16.75 (15.12–17.29)	0.091 (0.075–0.102)	0.911 (0.822–0.940)

*FA* Friedreich ataxia, *HC* healthy control

#### Antisaccades

The pooled data of leftward and rightward antisaccades were evaluated as well (Table 3). There was no remarkable difference between the groups with regard to peak velocities, latencies and durations of antisaccades. The incorrect ratios were higher in the *SYNE1* and FA patients than in the HC group. However, there was a mildly overlapping range in the 9.2° antisaccades within the *SYNE1* and HC subjects, whereas this was only minimally detected in the longer antisaccades (Fig. 3b).

**Table 3:** Antisaccade examination in *SYNE1* (AT-04–06) and Friedreich ataxia patients and in healthy controls

Subjects	9.2° antisaccades				18.4° antisaccades			
	Peak velocity (°/s)	Latency (s)	Duration (s)	Incorrect ratio	Peak velocity (°/s)	Latency (s)	Duration (s)	Incorrect ratio
AT-04	261.71	0.28	0.053	0.40	290.23	0.29	0.052	0.27
AT-05	232.76	0.32	0.072	0.64	280.16	0.37	0.097	0.36
AT-06	212.44	0.19	0.077	1.00	221.08	0.41	0.069	0.35
Median FA (range)	300.87 (237.22–351.78)	0.28 (0.19–0.41)	0.067 (0.059–0.072)	0.62 (0.28–0.96)	336.57 (253.63–439.47)	0.32 (0.20–0.45)	0.083 (0.062–0.103)	0.33 (0.06–1.00)
Median HC (range)	243.06 (197.67–313.18)	0.28 (0.23–0.33)	0.053 (0.046–0.075)	0.19 (0.08–0.54)	283.15 (209.63–382.10)	0.28 (0.24–0.37)	0.062 (0.049–0.088)	0.07 (0.00–0.29)

*FA* Friedreich ataxia,* HC* healthy control

#### Neuropsychological assessment

The neuropsychological assessment of FA and *SYNE1* patients are summarized in Table 4. The cognitive performance of ataxia patients was compared with the data of age- and education-matched standards in the literature [24–26]. Global cognition was only mildly reduced in two FA patients (AT-11 and AT-20), whereas the other subjects demonstrated normal ACE and MMSE scores. The LST results showed mild abnormalities in all *SYNE1* patients and in one FA patient, whereas the BDST results were decreased more prominently in both patient groups. These alterations indicate the impairment of working memory and in the ability to maintain and manipulate information. Surprisingly, the fluency test scores were in the normal range, only AT-04 patient demonstrated a mild deficit in the verbal fluency test. In addition, the RCFT results were equal to the standard outcomes, only AT-05 patient showed a mild impairment.

**Table 4:** Neuropsychological assessment of *SYNE1* and Friedreich ataxia patients

Patient code	Age (years)	Education (years)	ACE (93.7 ± 4.3)	MMSE (28.8 ± 1.3)	LST	BDST	Verbal fluency	Semantic fluency	RCFT copying	RCFT recall
*SYNE1* patients										
AT-04	35	14	89	29	2 (3.38 ± 0.79)^a^	4 (5.88 ± 1.1)^a^	10.5 (17.61 ± 5.42)^a^	14 (17.25 ± 3.96)	NA	NA
AT-05	43	14	93	29	2.33 (3.38 ± 0.79)^a^	3 (5.88 ± 1.1)^b^	14.5 (17.61 ± 5.42)	17 (17.25 ± 3.96)	35 (31.1 ± 3.6)	15 (23.7 ± 5.2)^a^
AT-06	37	12	89	29	2 (3.38 ± 0.79)^a^	2 (5.88 ± 1.1)^c^	16 (17.61 ± 5.42)	14 (17.25 ± 3.96)	36 (31.1 ± 3.6)	21.5 (23.7 ± 5.2)
Friedreich ataxia patients										
AT-08	23	12.5	93	30	3 (3.45 ± 0.89)	4 (5.88 ± 1.1)^a^	14.5 (16.13 ± 5.65)	11 (15.84 ± 4.51)	36 (31.1 ± 3.6)	24 (23.7 ± 5.2)
AT-11	57	16	87^a^	27	2.33 (3.11 ± 0.61)^a^	3 (5.34 ± 0.96)^b^	12 (11.02 ± 4.98)	16 (13.77 ± 4.05)	36 (29.2 ± 4.2)	26.5 (15.5 ± 5.5)
AT-12	60	17	95	29	3 (3.11 ± 0.61)	2 (5.34 ± 0.96)^c^	15.5 (11.02 ± 4.98)	20 (13.77 ± 4.05)	34 (29.2 ± 4.2)	27 (15.5 ± 5.5)
AT-20	16	10	88	26^a^	2.66 (3.33 ± 0.59)	5 (5.88 ± 0.96)	13.5 (13.83 ± 4.31)	14 (13.44 ± 3.52)	36 (31.1 ± 3.6)	26 (23.7 ± 5.2)
AT-21	59	14	94	30	3 (3.11 ± 0.61)	5 (5.34 ± 0.96)	15 (11.02 ± 4.98)	14 (13.77 ± 4.05)	32 (29.2 ± 4.2)	17.5 (15.5 ± 5.5)
AT-22	34	15	96	30	3.3 (3.38 ± 0.79)	4 (5.88 ± 1.1)^a^	16.5 (16.13 ± 5.65)	21 (15.84 ± 4.51)	34 (31.1 ± 3.6)	21 (23.7 ± 5.2)

In ACE and MMSE the lower normal threshold values of the normal population are in the brackets

In LST, BDST, verbal fluency, semantic fluency, RCFT copying and RCFT recall the age- and education-matched lower threshold values of the normal population are in the brackets

*ACE* Addenbrooke’s Cognitive Examination, *BDST* Backward Digit Span Task, *LST* Listening Span Task, *MMSE* Mini-Mental State Examination, *NA* not available, *RCFT* Rey Complex Figure Test

^a^Mild deficit, ^b^moderate deficit, ^c^severe deficit (Age- and education-matched standards, and standard deviations of the literature are delineated [23–25]. Mild, moderate and severe deficits mean that the cognitive impairment of the subject is more pronounced than one, two and three standard deviations of the normal standards, respectively)

## Discussion

In this paper we describe the clinical phenotype and characteristics of saccades and antisaccades of the first genetically confirmed Hungarian *SYNE1* patients caused by novel mutations. The cerebellar symptoms of these patients involved moderate to severe gait and lower limb ataxia and mild to moderate upper limb ataxia and dysarthria. Extracerebellar involvement was present as well, as all subjects have pyramidal signs and two of the three patients have some types of polyneuropathy. Moreover, AT-04 patient had strabismus, tactile sensitive myoclonic jerks and delayed puberty. In summary, the clinical phenotype of subjects is not purely cerebellar, in contrast to that described in the first French-Canadian population by Gros-Louis et al. [1], and similar to that of the later reported cases [2, 8]. This symptomatic variability suggests that *SYNE1* gene plays a broader role in the normal functioning of the nervous and musculoskeletal systems. Consequently, the mutations of this gene can cause symptoms and signs over a large spectrum, but an obvious genotype–phenotype correlation cannot be established [2].

The eye tracking examination revealed hypometric saccades in the 18.4° paradigm in all *SYNE1* patients and in two out of three in the 9.2° task. Saccadic dysmetria is a cerebellar symptom and it is a common eye movement abnormality in hereditary ataxias [27]. This is not a specific symptom for any type of inheritable ataxia, but there may be a higher proportion of hypo- or hypermetric saccades, serving as a supporting feature of the disease. The hypometria of *SYNE1* patients at large amplitude stimulus is more pronounced than the well-known mild hypometria in healthy subjects observed at higher target eccentricities [22]. The hypometria of *SYNE1* patients is presumably due to the involvement of the cerebellar oculomotor vermis and caudal fastigial nucleus [28]. In addition to accuracy, velocity is another important characteristic of saccades. Previous case reports described slowing of saccades in a portion of *SYNE1* patients, however these observations were based exclusively on physical examinations [1–4, 18, 19]. Our findings, obtained by fine eye tracking assessment, confirmed the clinical observations of some earlier publications, i.e., a high frequency of slow saccades can be detected in *SYNE1* ataxia. This lower saccadic velocity is likely due to brainstem involvement, in particular, the functional loss of pontine saccadic burst generator neurons and omnipause neurons can explain this observation [23]. Slowing of saccades is a characteristic eye movement abnormality in SCA2 disease. Federighi et al. examined the saccadic parameters of seven SCA2 patients at similar target eccentricities (10 and 18°) to those we used in this study and they found more severely reduced peak velocities and delayed saccadic latencies compared to controls than we detected in two of three *SYNE1* patients [23]. Presumably brainstem impairment is more pronounced in SCA2 than in *SYNE1*. In addition, saccadic hypometria was not found in SCA2 patients, whereas we observed lower saccadic amplitude in *SYNE1* patients compared to healthy subjects at the larger stimulus paradigm.

**Fig. 5:**
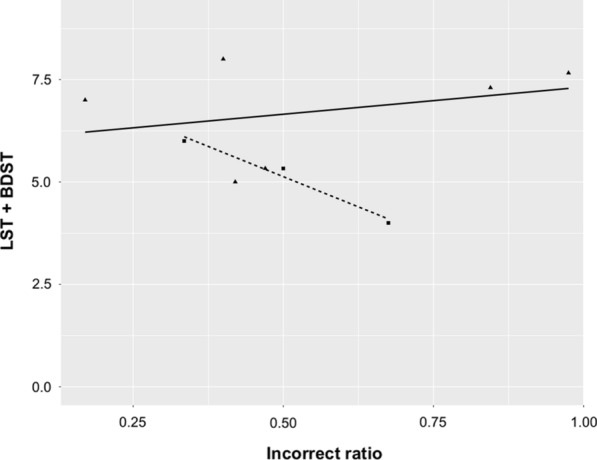
The delineation of the possible relationship between working memory test results and the incorrect ratio of antisaccades in ataxia patients. The horizontal axis denotes the mean value of the incorrect ratios of 9.2 and 18.4 antisaccade tasks, whereas the vertical axis indicates the sum of Listening Span Task (LST) and Backward Digit Span Task (BDST) scores; the triangles and squares indicate the data of Friedreich ataxia patients (FA) and *SYNE1* patients, respectively, and the regression line is drawn by continuous and dashed lines

The antisaccade assessment showed higher rates of incorrectly accomplished antisaccades in both FA and *SYNE1* patients compared to healthy subjects, whereas the other parameters were in similar ranges. The error rates were higher on the short stimulus amplitude task than on the long amplitude trial. Basically, target eccentricities affect gain, latency and peak velocity, whereas its influence on the incorrect ratios is not clear at these amplitudes [29–31]. The higher error rate raises the suspicion of cognitive impairment, because a strong correlation was demonstrated between antisaccades and working memory [32]. The neuropsychological assessment revealed that global cognitive performance was normal in *SYNE1* patients, whereas executive functions were impaired, especially working memory. The performance of the examined *SYNE1* subjects in BDST and LST paradigms inversely correlated with errors in the antisaccade tasks, i.e., the most severely affected patient in working memory tests (AT-06) had the highest error rate in the antisaccade paradigm (Fig. 5). A similar relationship was not detected in the FA group. Previously published studies indicated higher antisaccadic error rates in other hereditary and idiopathic cerebellar disorders, including ataxia with oculomotor apraxia type 1, 2, ataxia telangiectasia, SCA1, 2, 3 and late onset cerebellar ataxia (LOCA) [28, 33–35]. Additionally, Pretegiani et al. revealed that SCA2 and LOCA patients showed equally poor antisaccade performance irrespective of cortical involvement [33]. Additionally, a thorough investigation of a SCA2 patient cohort confirmed that impaired antisaccade efficacy was associated with executive test deficits, including Stroop interference task and verbal fluency test [34]. Our findings draw attention to the major role of working memory and inhibitory control in the performance of antisaccades, and confirm that executive dysfunction is a prevalent neuropsychological abnormality in hereditary ataxias as a part of the cerebellar cognitive and affective syndrome [36].

## Conclusions

In conclusion, this paper demonstrates the detailed neurological assessment of the first Hungarian *SYNE1* ataxia patients with novel pathogenic mutations. The eye tracking investigation detected some interesting alterations regarding both saccades and antisaccades in these subjects, including saccadic hypometria and increased error rates for antisaccades. The main weakness of this study is the low case number. Nevertheless, these pilot findings point out the importance of device-aided examination of eye movements in ARCAs. Hopefully in the near future, these parameters can be investigated in a larger number of *SYNE1* patients in order to be able to draw statistical conclusions as well.

## Data Availability

The datasets used and/or analysed in the current study are available in this paper.

## Abbreviations

ACE: Addenbrooke’s Cognitive Examination (ACE)
ARCA: Autosomal recessive cerebellar ataxias
ARCA1: Autosomal recessive cerebellar ataxia type 1
BDST: Backward Digit Span Task
FA: Friedreich ataxia
HC: Healthy controls
LOCA: Late onset cerebellar ataxia
LST: Listening Span Task
MMSE: Mini-Mental State Examination
NGS: New generation sequencing
RCFT: Rey Complex Figure Test
SARA: Scale for the Assessment and Rating of Ataxia
SCA: Spinocerebellar ataxia
SCAR8: Spinocerebellar ataxia, autosomal recessive 8
WES: Whole exome sequencing
