# Correction to: Eye-tracking-aided characterization of saccades and antisaccades in SYNE1 ataxia patients: a pilot study

**DOI:** 10.1186/s12868-021-00621-8

**Published:** 2021-03-10

**Authors:** Laszlo Szpisjak, Gabor Szaraz, Andras Salamon, Viola L. Nemeth, Noemi Szepfalusi, Gabor Veres, Balint Kincses, Zoltan Maroti, Tibor Kalmar, Malgorzata Rydzanicz, Rafal Ploski, Peter Klivenyi, Denes Zadori

**Affiliations:** 1 Department of Neurology, University of Szeged, Semmelweis u. 6, 6725 Szeged, Hungary; 2 Department of Psychiatry, University of Szeged, Szeged, Hungary; 3 Genetic Diagnostic Laboratory, Department of Pediatrics and Pediatric Health Center, University of Szeged, Szeged, Hungary; 4 Department of Medical Genetics, Medical University of Warsaw, Warsaw, Poland; 5 MTA-SZTE Neuroscience Research Group, Szeged, Hungary

## Correction to: BMC Neurosci (2021) 22:7 10.1186/s12868-021-00612-9

Following publication of the original article [1], the authors reported an error in Fig. 2b. The description of the mutation in the Intron 128–Exon 128 boundary is inappropriate as using the terminology for codons is restricted only for exons, and it cannot be applied at this site. Furthermore, the number of the intron preceding exon 128 should be marked as 127. Regarding the identified error the text itself needs the following minor correction in the second paragraph in page 7 of 12: ‘It causes a TAG–TGG codon change at the Intron 128–Exon 128 boundary resulting in an abnormal splicing variant (Fig. 2b).’ to the following ‘It causes an A>G change at the Intron 127–Exon 128 boundary resulting in an abnormal splicing variant (Fig. 2b).’

**Fig. 2:**
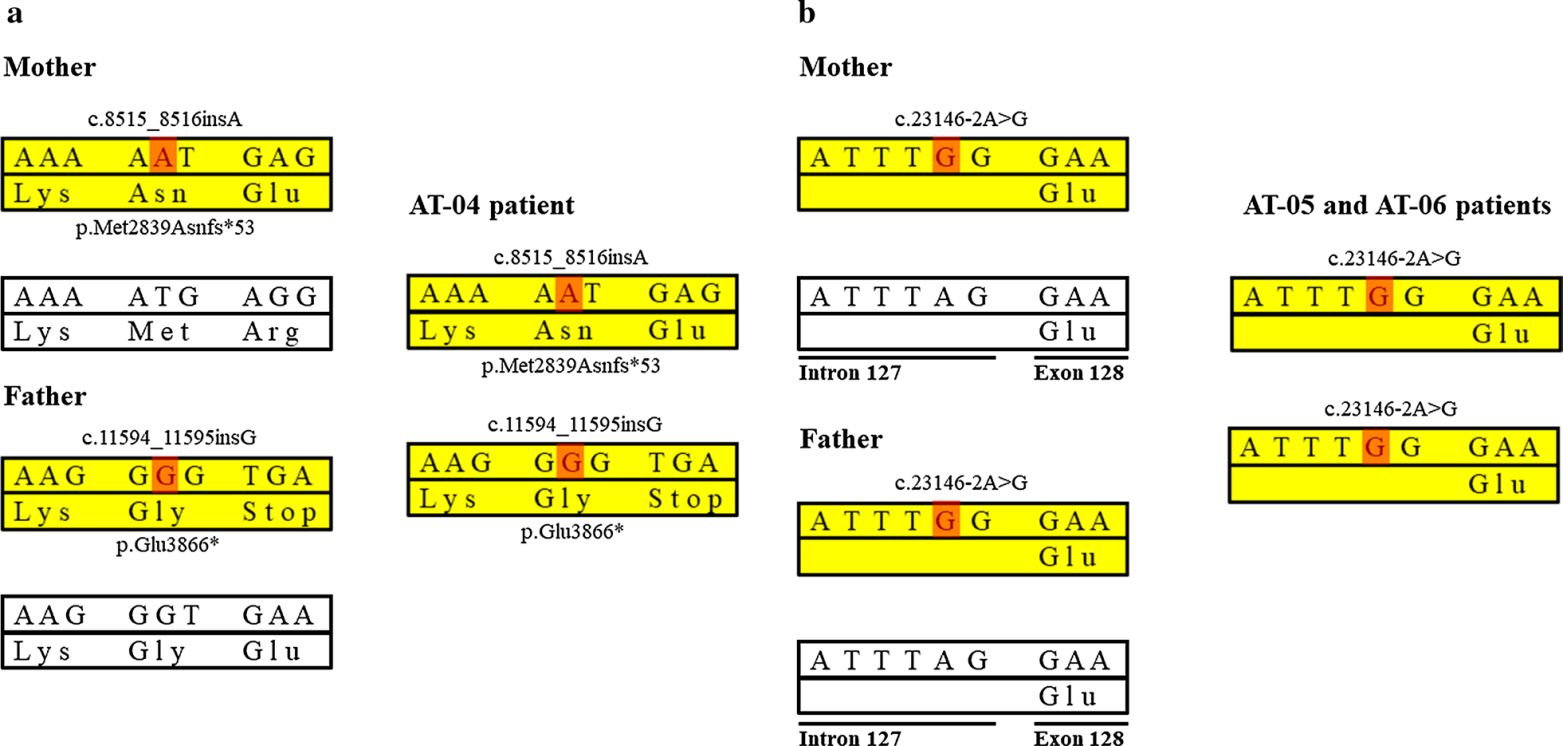
Genetic abnormalities and consequent alterations of protein of *SYNE1* ataxia patients and their parents. **a** *SYNE1* gene mutations in AT-04 patient and the parental origin of these variations. **b** *SYNE1* gene abnormalities in AT-05 and AT-06 subjects and the parental segregation of these mutations. The upper parts of the bars denote the DNA sequence, while the lower parts show the encoded amino acids of the protein. Yellow bars indicate the pathogenic alleles, white bands mark the normal alleles. Red highlights the nucleotide change of the *SYNE1* gene. In part **b**, the c.23146-2A>G mutation is located in the intron–exon boundary resulting in an abnormal splicing variant

The correct Fig. 2 is included in this Correction article, and the original article has been updated.
